# Accuracy of postvoid residual volumes after vaginal delivery: a prospective equivalence study to compare an automatic scanning device with transurethral catheterization

**DOI:** 10.1007/s00192-018-3700-9

**Published:** 2018-06-27

**Authors:** Femke E. M. Mulder, Sytske van der Velde, Fraukje Pol, Marjolein Bos, Jules Schagen van Leeuwen, Viviane Dietz, Robert A. Hakvoort, Jan-Paul W. R. Roovers

**Affiliations:** 1 Department of Obstetrics and Gynaecology, Academic Medical Centre, Meibergdreef 9 – room H4.240, 1105 AZ Amsterdam, The Netherlands; 2 Department of Obstetrics and Gynaecology, Spaarne Gasthuis, Haarlem, The Netherlands; 3 Department of Obstetrics and Gynaecology, Catharina Ziekenhuis, Eindhoven, The Netherlands; 4 Department of Obstetrics and Gynaecology, Antonius Ziekenhuis, Nieuwegein, The Netherlands; 5 Department of Obstetrics and Gynaecology, Martini Hospital, Groningen, The Netherlands

**Keywords:** Bladder scan, Postpartum, Urinary retention, Residual volume, Validation, Reliability

## Abstract

**Introduction and hypothesis:**

Abnormal postvoid residual volumes (PVRV) after delivery are common in daily clinical practice. By using an automatic scanning device, unnecessary catheterizations can be prevented. The aim of this study was to determine the accuracy of PVRV after vaginal delivery measured by an automatic scanning device through a comparison with transurethral catheterization.

**Materials and methods:**

This prospective observational equivalence study was performed in patients who delivered vaginally between June 2012 and May 2017 in three teaching hospitals in The Netherlands. After the first spontaneous void after delivery, postvoid residual volume (PVRV) was measured with a portable automatic scanning device (BladderScan® BVI 9400). Directly afterward, it was measured by catheterization. Correlation between measurements was calculated using Spearman’s correlation coefficient and agreement plot. The primary outcome was to validate the correlation between the BladderScan® compared with the gold standard of transurethral catheterization.

**Results:**

Data of 407 patients was used for final analysis. Median PVRV as measured by BladderScan® was 380 ml (± 261–0–999 ml) and by catheterization was 375 ml (± 315–1800 ml). Mean difference between measurements was −12.9 ml (± 178 ml). There was a very good correlation between methods (Spearman’s rho = 0.82, *p* < 0.001). Using a cut-ff value of >500 ml, specificity and sensitivity were 85.4 and 85.6%, respectively.

**Conclusions:**

The BladderScan® (BVI 9400) measures PVRV precisely and reliably after vaginal delivery and should be preferred over catheterization.

## Introduction

After delivery, abnormal postvoid residual volumes (PVRV) after spontaneous voiding attempts are encountered in up to 45% of women [1–5]. This condition is also known as asymptomatic or covert postpartum urinary retention (PUR). Although evidence for long-term clinical consequences of abnormal PVRV postpartum is not available, literature suggests that even a single episode of bladder overdistension can result in prolonged micturition problems, urinary tract infections (UTIs), and renal obstruction [6–9]. Therefore, accurate and timely diagnosis is important.

To measure PVRV, different techniques are available. Transurethral catheterization is considered the gold standard for accurate measurement of bladder volume and PVRV. However, real-time ultrasonography (US) has proven to be a valid and reliable substitute [10, 11]. Nevertheless, the use of a regular US device requires specific expertise and training and a mathematic calculation after measurement. This makes the method unsuitable for everyday clinical use. In order to facilitate the recognition of urinary retention, automatic portable devices have been developed. In many fields of medicine, screening for PVRV with a portable BladderScan® device is part of routine care, for example, in patients with neurogenic bladder, voiding dysfunction, or after orthopedic or urogynecological surgery [12–14]. In this way, unnecessary transurethral catheterizations can possibly be reduced [15, 16]. The effect of such a measure could be the reduction of nosocomial infections, patient discomfort, and healthcare costs. However, the technique is not widely accepted for postpartum women. A reason for this could be that the portable bladder scanner might not only measure bladder volume but also the (remaining bloody content of the) uterus and its pronounced vascularization. While the reliability of the BladderScan® is confirmed in men and nonpregnant women [17–19], its reliability in the puerperium remains inconclusive [20, 21]. Although several studies have compared portable bladder scanning devices with catheterization, these studies were either small or validation was done for relatively high residuals [4, 22–25]. To determine the accuracy of the BladderScan® in women directly after vaginal delivery, a prospective equivalence study was conducted.

## Methods

Between June 2012 and May 2017, this study was conducted in three Dutch teaching hospitals after approval by the medical committee of the Catharina Hospital in Eindhoven, The Netherlands (M11–1165), followed by the two other centers.

Women were asked to participate in this study prior to their delivery, if they were ≥ 18 years old and were to deliver vaginally. Women were either counselled and included before the onset of labor or were asked to participate after having given birth. Written informed consent was obtained from all patients.

### Outcomes

The primary outcome of this equivalence study was to determine the accuracy of the BladderScan® directly postpartum. To evaluate whether the BladderScan® can be used to detect abnormal or increased residual volumes in women after vaginal delivery, PVRV as measured by BladderScan® was compared with measurement by transurethral catheterization. Measurements were done directly after the first void after vaginal delivery. The agreement between these measurements was calculated using Spearman’s rank correlation coefficient with the following statistical cutoff values: 0–0.19 very weak; 0.20–0.39 weak; 0.40– 0.59 moderate; 0.60–0.79 strong, and 0.80 1.0 very strong correlations. In addition, an agreement plot was calculated. Second, we determined sensitivity and specificity of the device using the cutoff value of 500 ml, which we believe is a valid cutoff to discriminate between physiology and pathology, as described in a previous study [26]. We also calculated positive (PPV) and negative (NPV) predictive values.

### Measurements

PVRVs were measured with a portable bladder scanning device (BVI-9400 BladderScan®, Verathon Medical Europe, Ijsselstein, The Netherlands). After the first spontaneous micturition, bladder content was measured with the patient in the supine position. Scanning was performed abdominally two fingers above the symphysis pubis. Directly afterward, the bladder was emptied with a single-use catheter, and the residual volume was collected and measured. Prior to the use of the BladderScan®, nursing staff was trained by an expert from the manufacturer. In all hospitals, the same new model was used. The BladderScan® has an upper limit of 999 ml: cases in which the apparatus showed > 999 ml were considered and analyzed as being 999 ml. Pregnancy- and delivery-related data were obtained from electronic patient charts.

### Sample size calculation

The sample size was calculated based on equivalence between methods. With an alpha of 0.05, power of 80%, standard deviation (SD) of 50, and an expected difference of 10% (+5% and − 5%), we calculated that at least 310 paired measurements were necessary.

### Statistical analysis

Measurements of postvoid urinary volume by BladderScan® and catheterization were compared. For statistical analysis, SPSS version 24.0 (IBM) was used. Results are reported as mean (in case of normal distribution) or median (in case of abnormal distribution). To calculate the correlation, Bland-Altman plot, Spearman’s rank correlation coefficient, and Wilcoxon signed-rank tests were used when appropriate. A *p* value <0.05 was considered statistically significant.

## Results

Data of 407 patients could be used for final analysis. Table 1 shows patient characteristics.

**Table 1:** Patient characteristics

Patient variables	Results
Nulliparous	59% (*n* = 240)
Gestational age* (weeks)	39.7 (± 1.3)
Maternal age* (years)	30.5 (± 4.5)
Time first stage** (h)	5.8 (1–19)
Time second stage** (min)	34 (1–212)
Spontaneous vaginal delivery	79% (*n* = 317)
Assisted vaginal delivery	21% (*n* = 92)
Birth weight* (g)	3430 (± 469)
Any analgesia	66% (*n* = 262)
Epidural analgesia	44% (*n* = 181)
Vaginal rupture	42% (*n* = 173)
Episiotomy	49% (*n* = 191)
BMI**	24.2 (16–42)

*BMI* body mass index

*Mean (± standard deviation), **median (range)

Median volume of catheterization measurements was 375 ml (0–1800 ml), the 25th percentile was 150 ml, and the 75th percentile was 600 ml. The median volume of the BladderScan ® measurements was 380 ml (0–999 ml), with the 25th and 75th percentile being 200 ml and 567 ml, respectively. Mean difference between measurements was −13 ml (SD ± 177 ml). These results are presented in Table 2.

**Table 2:** Postvoid residual volume (PVRV) measurements

	Bladderscan	Catheterization
Minimum	0 ml	0 ml
Maximum	> 999 ml	1800 ml
Median (± SD)	380 ml (± 261)	375 ml (± 315)
Mean Difference (± SD)	−12,87 ml (± 178)	

*SD* standard deviation

**Fig. 1:**
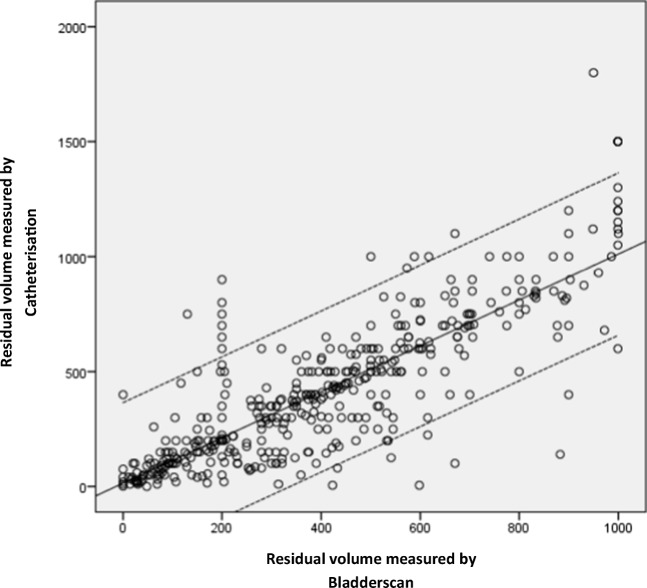
Correlation between residual volume measured by BladderScan and catheterization (*dashed lines* 95% confidence interval)

Agreement between measurement methods are presented in Fig. 1.

**Fig. 2:**
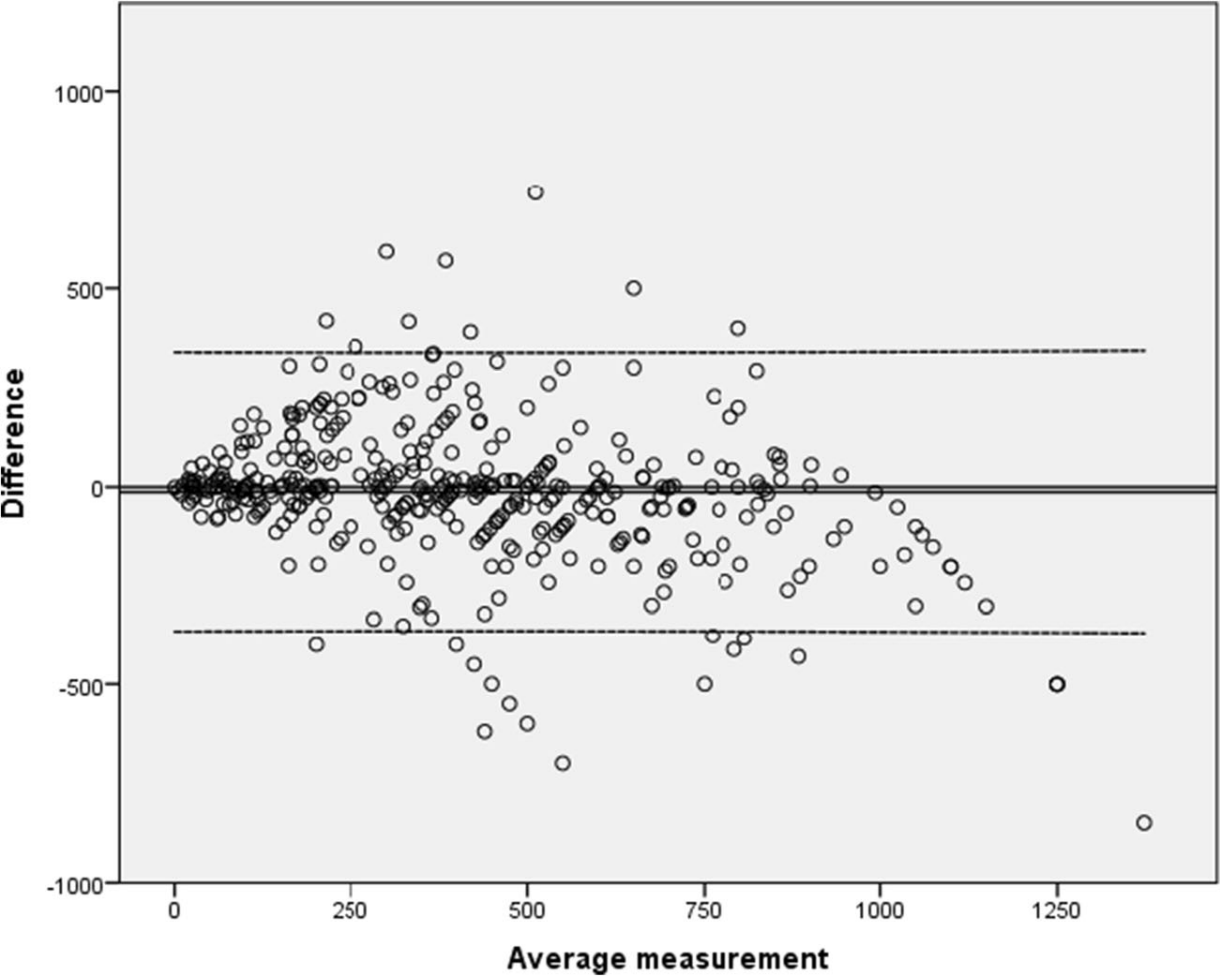
Agreement plot (95% limits of agreement)

Spearman’s rank correlation coefficient calculation showed a close correlation between BladderScan® and catheterized bladder volumes (*r* = 0.82, *p* < 0.01). Spearman’s rank correlation coefficient was repeated after excluding extreme data (i.e., BladderScan > 999 ml only and BladderScan > 999 ml plus catheterization volume >1000 ml.) With these restrictions, outcomes remained similar, resulting in close correlations of *r* = 0.80 and *r* = 0.78, respectively. An agreement plot with 95% confidence interval (CI) showing mean average of each measurement compared with the difference between measurements is shown in Fig. 2. Here, a good agreement over the whole measurement range of 0–1000 ml was found.

Table 3 shows mean PVRV by BladderScan® and catheterization and mean difference between the two categorized per 100-ml group (as measured with a BladderScan®).

**Table 3:** Median differences per category (per 100 ml)

BladderScan category per 100 ml	No. patients	Median PVRV BladderScan (± SD)	Median PVRV catheterization (± SD)	Median difference (± SD)
0–100 ml	56	53 ml (±31)	60 (±68)	−7 (±66)
101–200 ml	60	164 ml (±33)	195 (±211)	−12 (±200)
201–300 ml	44	263 ml (±32)	212 (±132)	12 (±130)
301–400 ml	56	350 ml (±29)	350 (±139)	2 (±131)
401–500 ml	59	450 ml (±31)	450 ml (±164)	0 ml (±156)
501–600 ml	46	551 ml (±29)	575 ml (±211)	−14 ml (±202)
601–700 ml	31	667 ml (±31)	690 ml (±210)	0 ml (±205)
701–800 ml	16	767 ml (±39)	800 ml (±137)	−51 ml (±140)
801–900 ml	20	877 ml (±32)	830 ml (±228)	17 ml (±232)
901–999 ml	5	960 ml (±37)	875 ml (±147)	56 ml (±178)

*SD* standard deviation

Table 4 shows sensitivity and specificity calculated using a PVRV ≥500 ml as a cutoff value. PPV and NPV were 91.9% and 75.3%, respectively.

**Table 4:** Postvoid residual volume sensitivity and specificity ≥500 ml

	Catheterization < 500 ml	Catheterization ≥ 500 ml	Total (*n*)
BladderScan < 500 ml	*n* = 229 Specificity 85.4%	*n* = 39	*n* = 268
BladderScan ≥ 500 ml	*n* = 20	*n* = 119 Sensitivity 85.6%	*n* = 139
Total	*n* = 249	*n* = 158	*n* = 407

## Discussion

Based on our study results, we conclude that the BladderScan® is an accurate and therefore valid alternative to the invasive measurements obtained by catheterization in measuring PVRV directly after vaginal delivery. By measuring PVRVs in a large and nonselected group of women after vaginal delivery, a high degree of agreement was found between the two measurement methods. This agreement was found in the whole range of PVRVs, which is known to be very wide directly postpartum [5, 27]. We believe this is sufficient evidence to support the use of the BladderScan® BVI-9400 after vaginal delivery. Measuring PVRV is not part of standard postpartum care, and various authors have shown that increased residual volumes often normalize within several days [27, 28]. However, in daily clinical practice, there is a need to identify abnormal PVRV—for instance, when micturition produces very small portions of urine.

To minimize the risk of damage to the bladder, accurate and timely measurement of PVRV is important to determine whether treatment should be started. In the absence of other techniques, and because of a slight advantage that catheterization is a measurement technique as well as a treatment, it has been the accepted gold standard for PVRV measurements. While it can cause considerable physical discomfort and inevitably increases the risk for UTIs, noninvasive alternatives are nevertheless preferable.

Evidence regarding accuracy of noninvasive methods after delivery is scarce. However, concerning automatic scanning devices, seven earlier studies reported their validity or reliability [4, 20–25]. Most of these studies reported on a small patient sample and in selected cases [21, 24, 29]. Our study had a considerably larger sample size and was prospectively designed as an equivalence study to objectively assess accuracy of the BladderScan®. Further, this study was conducted in an unselected cohort of women, whereas other studies were potentially biased by including women at risk of having abnormal PVRV, who were only catheterized in case PVRV >300 ml, or with an indwelling Foley catheter in situ, which was clamped to measure bladder volumes [21, 24, 25]. This inevitably leads to selection bias and therefore validation of the BladderScan® only in women with higher residual volumes.

Furthermore, PVRV of patients in our study were measured using the same, newer, model, i.e. BVI 9400. To our knowledge, this study is the first to determine the accuracy of this newer model. Previous models (BVI 3000 and BVI 6100) were, according to the manufacturer, inferior in discriminating bladder volumes from the uterus itself.

A recurring thought is that an automatic scanning device could overestimate residual volumes, as it would also measure the content of the uterus. However, others demonstrated by repetitive US after delivery that in the early puerperium, the cavity of the uterus is generally empty, and fluid and debris are mostly seen in the middle puerperium (from the 7th day after delivery) [30]. In addition, median volume of the uterus after delivery is 22 cm^3^. This shows that the thoughts on overestimation of PVRV by the BladderScan® after delivery are not based on evidence and are therefore unfounded [31].

Nevertheless, there are some limitations to our study that need to be addressed. First, a large group of nurses performed the measurements. Despite the ease of use and the structured training they all received before the study commenced, this still may have introduced variation in measurements. While we have no data on measurements recorded by different persons measuring for this study, interobserver coefficients cannot be reproduced. Potentially, with a smaller nursing staff or more extensive training, performance of the BladderScan ® would have been better. However, despite this, we believe measurements done by a larger group of nurses reflects daily practice. Further, the effect of an interobserver variation is expected to be small, as a very strong correlation was found between measurements. The second limitation is that the time between measurement with the BladderScan® and catheterization was often not recorded in patient files. Both transurethral catheterization and scanning will only produce reliable values when measurements are performed directly after micturition to minimize refilling due to continuing diuresis [32, 33]. Although by protocol measurements had to be performed directly after each other, we do not have data indicating whether the protocol was followed in all cases. This would have been especially interesting in cases in which extreme differences were found between the two techniques, possibly resulting in a better correlation.

The importance of any measurement technique is that it is accurate in discriminating between patients who need to be treated and who do not. We have shown that this type of scanning device is suitable for all volume ranges. However, to minimize overtreatment, a valid cutoff value to discriminate physiology from pathology is essential. In the literature, asymptomatic (covert) PUR is defined as a PVRV ≥150 ml after spontaneous micturition. Previously, we suggested increasing this cutoff value to ≥500 ml based on the high residual volumes found in a previous unselected cohort of women after vaginal delivery [5]. With a median PVRV of 375–380 ml found in this study (±216–315 ml), we believe that the commonly used limit of 150 ml lies well within the physiological range and probably results in unnecessary management of a physiological phenomenon. Using the new cutoff value of PVRV ≥500 ml, we believe the BladderScan® can accurately be used in clinical daily practice, with a specificity of 85.4% and sensitivity of 85.6%. (Table 4).

## Conclusion

The BladderScan® BVI 9400 is an accurate alternative to evaluate PVRV after vaginal delivery and is preferred over transurethral catheterization to reduce the risk of UTI and optimize patient comfort.
